# Lactylation Modification: From Basic Biological Process to Clinical Cardiovascular Diseases

**DOI:** 10.34133/research.1228

**Published:** 2026-04-03

**Authors:** Jilong Geng, Zheyan Fang, Zhentao Zhang, Junbo Ge, Hua Li

**Affiliations:** ^1^ Department of Cardiology, Zhongshan Hospital, Fudan University, Shanghai 200032, China.; ^2^ Shanghai Institute of Cardiovascular Diseases, Shanghai 200032, China.; ^3^ State Key Laboratory of Cardiology, Shanghai 200032, China.; ^4^ National Clinical Research Center for Interventional Medicine, Shanghai 200032, China.; ^5^ Shanghai Clinical Research Center for Interventional Medicine, Shanghai 200032, China.; ^6^ Key Laboratory of Viral Heart Diseases, National Health Commission, Shanghai 200032, China.; ^7^ Key Laboratory of Viral Heart Diseases, Chinese Academy of Medical Sciences, Shanghai 200032, China.

## Abstract

Cardiovascular diseases (CVDs) remain the leading cause of death worldwide and are hallmarked by profound disturbances in energy metabolism and maladaptive tissue remodeling. Lactate, long dismissed as a metabolic waste product, is now recognized as a context-dependent central carbon source and signaling metabolite. A key recent advance is the discovery of lysine lactylation (Kla), an evolutionarily conserved posttranslational modification that couples lactate abundance to chromatin state and protein function. Here, we synthesize current knowledge on the biogenesis and enzymatic regulation of Kla, and delineate how lactate-driven histone and nonhistone lactylation remodel transcriptional and signaling networks controlling fibrosis, energy metabolism, immune and inflammatory responses, and angiogenesis. We then focus on emerging evidence that Kla is a nodal regulator across major cardiovascular pathologies—including atherosclerosis, myocardial infarction and ischemia/reperfusion injury, heart failure, valvular and arterial calcification, and pulmonary hypertension—where it can act as a context-dependent “accelerator” or “brake” of disease progression. Finally, we outline a translational framework that targets the lactate–lactylation axis at 3 levels: lactate transport, lactate production, and lactylation writers/erasers, highlighting opportunities and challenges for therapeutic intervention. Together, these insights position protein lactylation as a pivotal metabolic–epigenetic interface in the cardiovascular system and a promising entry point for precision therapies in CVDs.

## Introduction

Lactate, long regarded simply as a metabolic byproduct of anaerobic glycolysis, is now recognized as a crucial metabolite with diverse signaling functions that modulate cellular processes through multiple pathways [1,2]. A major recent advance in lactate research is the identification of lactylation, a novel posttranslational modification (PTM) in which lactyl groups are covalently attached to lysine residues on histone and nonhistone proteins [3]. This modification provides a direct biochemical connection between cellular metabolic states and transcriptional regulation, offering new mechanistic insights into how metabolic intermediates shape gene expression programs and cellular behavior.

Lactylation was initially identified on histones, where it was shown to directly regulate chromatin structure and gene transcription [4]. Subsequent studies revealed that this acylation also occurs on a broad spectrum of nonhistone substrates, thereby influencing diverse biological processes, including immune responses, fibrosis, and cellular differentiation [5]. The dynamic regulation of this modification is mediated by enzymatic “writers”, “readers”, and “erasers” and is tightly coupled to glycolytic flux and the availability of intracellular lactate. As new experimental data continue to emerge, these advances have substantially deepened understanding of how metabolic signals induce persistent changes in cellular phenotypes.

Cardiovascular diseases (CVDs), including myocardial infarction (MI), heart failure, and atherosclerosis, are characterized by metabolic reprogramming, immune activation, and tissue remodeling—processes that intersect with lactate accumulation and lactylation dynamics [6]. Mounting evidence suggests that protein lactylation is a key regulatory node in CVDs, modulating endothelial homeostasis, macrophage polarization, fibrotic remodeling, and neovascularization [7]. Collectively, these findings support the notion that lactylation functions as both an “accelerator and brake” across major cardiovascular pathologies. However, with the rapidly expanding body of research on lactylation, rigorous synthesis and critical appraisal of these data have become increasingly necessary. Particularly when conflicts arise among findings across different disciplines, systematic integration and critical analysis are required. In this review, recent advances in lactylation research are systematically summarized, with particular emphasis on elucidating its multifaceted roles in cardiovascular homeostasis and disease. The aim of this review is to refine current conceptual frameworks of lactylation and to propose plausible therapeutic strategies targeting this modification in CVD.

## Lactate Metabolism and Lactylation Regulation

**Fig. 1.**
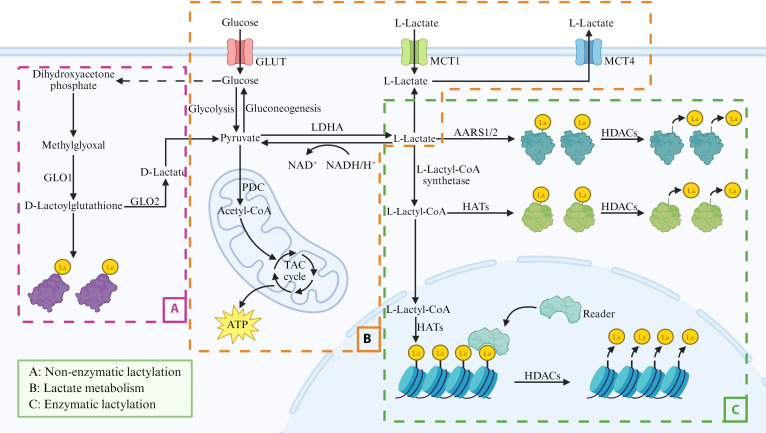
Lactate metabolism and lactylation in cells. Intracellular l-lactate is either produced via glycolysis or transported into cells from the extracellular environment through monocarboxylate transporters. l-Lactate can participate in enzymatic lactylation either directly or after being converted into lactyl-CoA. Furthermore, d-lactoylglutathione, generated through the glyoxalase pathway, can contribute to nonenzymatic lactylation. Created in BioRender. 1, 1. (2026) https://BioRender.com/x8piopa.

Lysine lactylation (Kla) consists of 2 stereoisomeric variants, KD-la and KL-la (Fig. 1). Recent studies indicated that KL-la is the predominant Kla variant [8,9]. This modification has been observed on both histone and nonhistone substrates, and its abundance is modulated by fluctuations in glycolytic flux. In view of its predominance and regulatory relevance, the present discussion primarily focuses on KL-la. In contrast, KD-la is generated nonenzymatically through the reaction of lysine residues with lactoylglutathione (LGSH), without requiring enzymatic catalysis [10] (Fig. 1A). Elucidation of the mechanisms underlying Kla is essential for a better understanding of its role in disease development, particularly in cardiovascular disorders.

### Lactate metabolism

#### Lactate production

Under physiological conditions, lactate production increases when cellular adenosine triphosphate (ATP) demand exceeds the capacity of oxidative phosphorylation (OXPHOS), such as during intense exercise or hypoxia [11]. As a key metabolic intermediate, lactate is predominantly produced from pyruvate generated by glycolysis (Fig. 1B). While pyruvate can be directed into mitochondrial oxidation under normoxia, it is reduced to lactate by cytosolic lactate dehydrogenase A (LDHA) under hypoxia [12,13]. Notably, the conversion of pyruvate to lactate also occurs under aerobic conditions, even when oxygen is abundant [14].

#### Lactate clearance

Excess lactate accumulation can be detrimental and may lead to lactic acidosis [15]. However, lactate clearance does not imply disposal; rather, it reflects metabolic recycling and conversion into other usable substrates. The reutilization of lactate primarily proceeds via 2 metabolic pathways (Fig. 1B). Lactate is oxidized back to pyruvate by lactate dehydrogenase B (LDHB), thereby supporting oxidative energy metabolism in high-demand organs such as the heart and brain [16]. Alternatively, lactate undergoes gluconeogenic conversion, predominantly in hepatic and renal tissues, via the Cori cycle to replenish blood glucose levels [17]. Collectively, these coordinated pathways prevent the toxic accumulation of lactate and help maintain cellular energy homeostasis.

#### Lactate transport

The transport of lactate across cell membranes is primarily mediated by proton-coupled monocarboxylate transporters MCT1 to MCT4 (SLC16 family) [18,19]. They enable the bidirectional transport of lactate driven by the combined concentration gradients of lactate and protons [20]. Among them, MCT1 and MCT4 serve as the principal mediators of lactate transport (Fig. 1B). MCT1, ubiquitously expressed, predominantly facilitates lactate uptake into oxidative cells (e.g., cardiac myocytes, slow-twitch muscle fibers, and hepatocytes), thereby supporting aerobic metabolism and gluconeogenesis [21]. In contrast, MCT4, enriched in glycolytic cells (e.g., fast-twitch muscle fibers), is mainly responsible for lactate efflux [19,22]. This functional divergence forms the basis of the lactate shuttle hypothesis [23,24]. MCT2 and MCT3 display more restricted expression patterns (e.g., neuron and retinal pigment epithelia, respectively) [25,26]. In epithelial barriers, sodium-coupled monocarboxylate transporters SMCTs (SLC5 family) can additionally contribute to lactate uptake/reabsorption, particularly in the intestine and kidney [27]. Collectively, these diverse roles position MCTs/SMCTs as a sophisticated network critical for lactate transport, metabolic integration, and the preservation of tissue and cellular energy homeostasis.

### The regulation of lactylation

#### Lactate activation to lactyl-CoA

The lactylation pathway is initiated by the metabolic conversion of lactate to its high-energy thioester, lactyl-coenzyme A (CoA). This activated conjugate serves as the requisite donor of lactyl groups to target proteins. It has been demonstrated that acetyl-CoA synthetase 2 (ACSS2) functions as a bona fide lactyl-CoA synthetase, catalyzing the formation of this metabolite [28]. Another nuclear-localized enzyme, guanosine triphosphate (GTP)-specific succinyl-CoA synthetase (GTPSCS), has also been shown to generate lactyl-CoA [29]. The lactyl-CoA pool generated through these pathways functions as a critical link that integrates metabolic signals to regulate a broad spectrum of cellular activities through the modification of diverse protein substrates.

#### Dynamic regulation of lactylation

PTMs are regulated by 3 classes of enzymes: writers, readers, and erasers [30]. Similarly, the spatiotemporal control of Kla requires the coordinated action of these enzymes (Fig. 1C). Lactyltransferases (writers) transfer lactyl groups to lysine residues. Among these lactyltransferases, histone acetyltransferases (HATs) represent major enzymatic contributors, with key representatives spanning the p300/CBP [31], GNAT (e.g., GCN5) [32], and MYST (TIP60, HBO1, MOF, and ESCO2) [33–36] families. Beyond canonical HATs, other acetyltransferase-related enzymes including DLAT [37] and ACAT [38] also participate in mediating Kla. Furthermore, alanyl-tRNA synthetases (AARS1/2) act as ATP-dependent lactyltransferases that couple lactate and ATP to globally modulate the lactylome [39]. Lactylation is interpreted by lactylation-specific reader proteins. For example, BRG1 binds H3K18 lactylation (H3K18la) via its bromodomain to promote mesenchymal–epithelial transition [40]. In addition, DPF2 (a subunit of the BAF complex) interacts with H3K14 lactylation (H3K14la) through its DPF domain [41]. Delactylation is mediated by deacylases (Erasers) including class I histone deacetylases (HDAC1 to HDAC3 and HDAC8) [42,43] and sirtuins (SIRT1 to SIRT3) [44,45]. Recent studies further suggest that SIRT6 functions as a histone delactylase; however, whether it also participates in nonhistone delactylation remains to be determined [46]. Together, these coordinated writer, reader, and eraser activities ensure dynamic and context-dependent regulation of the Kla landscape. Dissecting this enzymatic network is crucial for understanding how lactate-driven signaling shapes gene expression and may reveal new therapeutic targets in cardiovascular and other diseases.

## Molecular Function of Lactylation

**Fig. 2.**
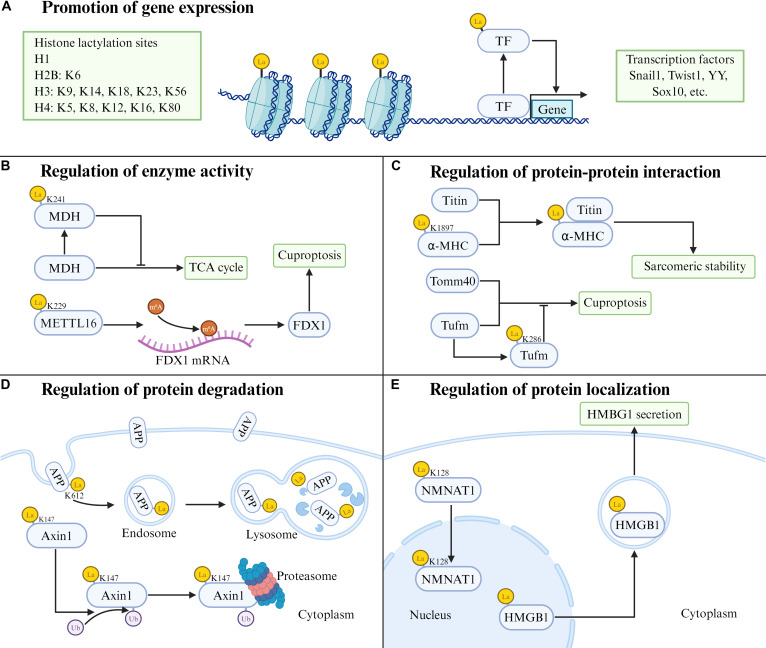
Molecular functions of lactylation. Lactylation of histones and transcription factors promotes gene expression (A). Lactylation of nonhistone proteins modulates protein functions through multiple mechanisms, including the regulation of enzyme activity (B), protein–protein interactions (C), protein degradation (D), and protein localization (E). Created in BioRender. 1, 1. (2026) https://BioRender.com/ua3vvp5.

As research progresses, an increasingly broad range of functional roles associated with lactylation is being uncovered. In general, histone lactylation primarily acts at the level of gene transcription, modulating the expression of specific target genes. By contrast, lactylation of nonhistone proteins influences a broad spectrum of functional properties, including catalytic activity, conformational or structural stability, intracellular localization and trafficking, and the capacity to interact with other macromolecules within the cell (Fig. 2).

### Histone lactylation regulates gene expression

Histone lactylation represents a novel epigenetic mark that activates chromatin and stimulates transcription of target genes. This modification functions analogously to established histone PTMs such as methylation, phosphorylation, and acetylation, all of which regulate gene expression by remodeling nucleosome architecture [47] (Fig. 2A). Lactylation has been identified at multiple lysine residues across histones H1, H2B, H3, and H4, with potential implications for gene regulatory networks and disease pathogenesis [48–50].

Current research has predominantly focused on the lactylation site H3K18, whereas other identified sites (e.g., H3K9 and H4K12) remain poorly characterized. Von Meyenn and colleagues [51] generated genome-wide datasets demonstrating the prevalence of H3K18la across diverse cell types and tissues. This epigenetic mark localizes to both active promoters—particularly those enriched for CpG islands—and tissue-specific enhancers [51]. H3K18la levels exhibit positive correlations with gene expression and with other active histone marks. Key transcriptional targets of H3K18la include leucine-rich alpha-2-glycoprotein 1 (LRG1), vascular endothelial growth factor A (VEGFA), interleukin-10 (IL-10) [52], major zygotic genome activation (ZGA) genes [53,54], POM121 [55], and VCAM1 [56]. Histone lactylation is increasingly recognized as a contributor to CVD, with H3K18la implicated in atherosclerosis [57], MI [58], heart failure [59], arterial calcification [60], and aortic aneurysm/dissection [61]. In addition, lactylation of H3K9 and H4K12 (H3K9la, H4K12la) has also been reported to promote atherosclerotic development [62,63]. In summary, further elucidation of the full spectrum of lactylation sites and their gene-specific transcriptional outcomes will be essential for clarifying disease mechanisms and for exploring histone lactylation as a potential therapeutic target.

### Nonhistone lactylation alters protein function

#### Lactylation-mediated regulation of catalytic activity

The effects of lactylation on enzymatic catalytic activity vary across different conditions (Fig. 2B). Specifically, lactylation can exert inhibitory effects on enzyme function in certain pathological contexts. For instance, lactylation of malate dehydrogenase 2 (MDH2) at Lys^241^ during myocardial ischemia/reperfusion (I/R) injury inhibits its catalytic function in the tricarboxylic acid (TCA) cycle [64]. Additionally, during aortic dissection, ATP5F1A (ATP synthase subunit) undergoes K531 lactylation, which impairs ATP synthase activity [65]. By contrast, lactylation can also enhance the catalytic activity of enzymes. Lactylation of METTL16 at K229 augments its ability to methylate FDX1 mRNA, which can induce cuproptosis by stabilizing FDX1 [45]. In sum, lactylation-mediated regulation of catalytic activity exhibits a clear duality: It can either enhance or inhibit enzyme function, with these divergent outcomes closely linked to the specific enzyme targets and the environmental contexts in which they operate.

#### Lactylation-mediated protein–protein interactions

Lactylation directly modulates protein–protein interaction patterns, not only disrupting existing interactions but also enhancing them or creating new ones (Fig. 2C). The p300-catalyzed and SIRT1-reversed lactylation of α-myosin heavy chain (α-MHC) at Lys^1897^ enhances its interaction with Titin, thereby maintaining sarcomeric stability and supporting cardiac structure and contractile function [66]. In radiation-induced heart disease (RIHD), K311 lactylation of protein disulfide-isomerase (P4HB) promotes its binding to prostaglandin G/H synthase 2 (PTGS2), thereby driving RIHD-associated pathogenic signaling [67]. In contrast, lactylation can also disrupt protein–protein interactions (PPIs) that are critical for cellular homeostasis. Lactylation of Tufm at Lys^286^ reduces its interaction with Tomm40, thereby compromising mitophagy and contributing to neuronal apoptosis in traumatic brain injury [68]. Collectively, these findings demonstrate that lactylation functions as a versatile molecular switch that remodels protein–protein interaction networks in a context-dependent manner, thereby fine-tuning essential cellular processes while also predisposing to pathology when dysregulated.

#### Lactylation-mediated control of protein degradation

Lactylation exerts a bidirectional regulatory role—either promoting or inhibiting protein degradation—by directly modifying key molecules in degradation pathways (Fig. 2D). The primary regulatory routes involve protein degradation via the ubiquitin–proteasome system or lysosomal clearance. Within proteasome-dependent degradation, lactylation of Axin1 at Lys^147^ decreases its stability by enhancing ubiquitination and targeting the protein for proteasomal breakdown [69]. Conversely, lactylation can impair ubiquitination, as exemplified by Serpina3k lactylation at Lys^351^ [70]. In cardiac fibroblasts, lactylated Serpina3k displays a markedly prolonged half-life, and its turnover is largely abolished by the proteasome inhibitor MG132, supporting lactylation-mediated suppression of proteasome-dependent degradation [70]. In lysosome-dependent degradation, lactylation can redirect protein trafficking to lysosomal pathways, with Alzheimer’s disease (AD) providing a notable example [71]. Specifically, lactylation of amyloid precursor protein (APP) at Lys^612^ modulates its metabolism, reducing amyloid-β (Aβ) production in AD. This modification shifts APP trafficking away from BACE1-mediated cleavage, directing it instead toward CD2AP-dependent endosomal–lysosomal degradation [71]. Ongoing investigation of this bidirectional regulation of protein degradation by lactylation is expected to clarify how diverse metabolic states orchestrate protein homeostasis.

#### Lactylation-mediated regulation of subcellular localization

Lactylation can affect intracellular protein localization by altering physicochemical properties or the efficiency of signal recognition between proteins and transport molecules (Fig. 2E). Specifically, p300-mediated lactylation of ABCF1 at Lys^430^ induces conformational changes that expose the C-terminal nuclear localization signal (NLS), thereby facilitating its nuclear translocation [72]. Additionally, in neutrophils following myocardial I/R injury, S100a9 lactylation at Lys^26^ facilitates its importin β1-dependent nuclear translocation, where S100a9 functions as a transcriptional coactivator to up-regulate migration/adhesion-related genes [37]. In contrast, p300/CBP-mediated lactylation triggers the translocation of HMGB1 to the cytoplasm, facilitating its extracellular secretion via exosomes [73,74]. Similarly, lactylation drives the nuclear-to-cytoplasmic translocation of CIRP, followed by its release into the extracellular milieu during sepsis. This extracellular CIRP then enables macrophages to regulate pulmonary vascular endothelial cell (PVEC) death [75]. Collectively, lactylation dynamically regulates the subcellular localization of proteins, enabling them to adapt to the physiological and pathological needs of cells.

#### Lactylation-mediated regulation of gene expression (via nonhistone factors)

Transcriptional regulation represents a primary function of lactylation. This process not only modulates gene expression by altering chromatin architecture but also directly modifies transcription factors, thereby regulating the expression of key proteins implicated in various diseases [76,77]. Lactylation of Snail1 and Twist1 enhances transcriptional activity, leading to increased transforming growth factor-β1 (TGF-β1) expression [78,79]. In atherosclerosis, Sox10 lactylation activates transcriptional programs that promote vascular smooth muscle cell (VSMC) transdifferentiation [80]. Therefore, lactylation serves as a direct and potent modulator of transcriptional networks, governing gene expression by targeting both chromatin and specific transcription factors across diverse physiological and pathological contexts.

## Biological Processes Modulated by Lactylation

### Lactylation in fibrosis

**Fig. 3.**
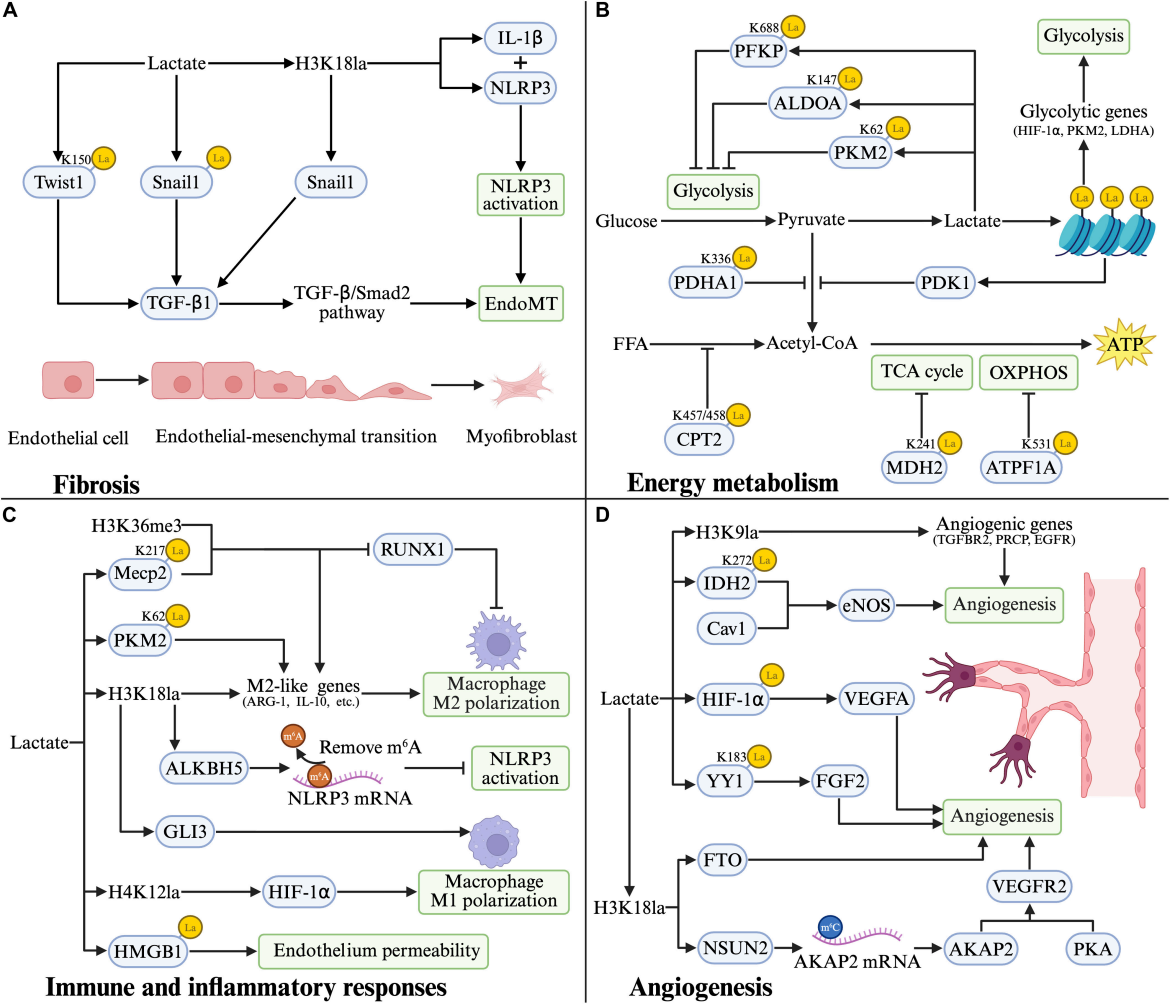
Lactylation links metabolic rewiring to cardiovascular-relevant cellular programs. (A) Fibrosis. Increased lactate promotes H3K18 lactylation and lactylation of key EndoMT transcription factors (e.g., Twist1 and Snail1), reinforcing TGF-β production and downstream TGF-β/Smad2 signaling to drive EndoMT and myofibroblast formation. H3K18la also enhances IL-1β expression and NLRP3 inflammasome activation, further facilitating EndoMT. (B) Energy metabolism. Lactylation modulates glucose metabolism and mitochondrial energy production by modifying glycolytic enzymes (PFKP, ALDOA, and PKM2), pyruvate oxidation (PDHA1 and PDK1), fatty acid oxidation (CPT2), and mitochondrial TCA/OXPHOS components (MDH2 and ATP5F1A), collectively reprogramming cellular energy metabolism. (C) Immune and inflammatory responses. On the anti-inflammatory side, increased lactylation supports M2-like polarization by inducing M2-associated genes (e.g., ARG1 and IL-10) and attenuates NLRP3 inflammasome activation by regulating NLRP3 mRNA stability. Conversely, lactylation can also potentiate inflammatory signaling by promoting M1 polarization and by enhancing endothelial barrier dysfunction, thereby increasing endothelial permeability. (D) Angiogenesis. Lactylation promotes angiogenesis by enhancing angiogenic gene expression via H3K9 lactylation and by engaging lactylation-dependent signaling nodes, including the IDH2–Cav1–eNOS, HIF-1α–VEGFA, and YY1–FGF2 axes. In addition, H3K18 lactylation increases FTO to support pro-angiogenic programs and induces NSUN2 to up-regulate AKAP2, thereby activating the AKAP2/PKA–VEGFR2 signaling pathway and driving neovascularization. Created in BioRender. 1, 1. (2026) https://BioRender.com/45qyz5w.

Fibrosis, characterized by excessive extracellular matrix (ECM) deposition, is a shared end-stage feature across major CVDs, including fibrous cap formation in atherosclerotic plaques, post-ischemic ventricular remodeling, heart failure progression, and hypertensive vascular remodeling [81]. Fibrosis-driven remodeling is a central determinant of cardiac stiffness, ventricular dysfunction, and adverse outcomes after myocardial injury [82]. Emerging evidence supports lactylation as a metabolic–epigenetic switch that reshapes chromatin states and transcription factor activity toward a pro-fibrotic program (Fig. 3A). In vascular and cardiac remodeling contexts, endothelial-to-mesenchymal transition (EndoMT) represents a key fibrogenic route: In atherosclerosis, increased H3K18la has been associated with up-regulated Snail1 expression, thereby promoting EndoMT [57]. After MI, lactylation of Snail1 activates TGF-β1 transcription and the downstream TGF-β/Smad2 signaling pathway, thereby driving EndoMT and exacerbating fibrotic progression [78]. Similar to Snail1, Twist1, another pro-EndoMT transcription factor, has also been reported to undergo lactylation, thereby promoting EndoMT by enhancing TGF-β1 expression [79]. In addition, recent evidence from pulmonary fibrosis demonstrated that lactate-induced H3K18la suppresses the ubiquitin ligase carboxyl terminus of Hsc70-interacting protein (CHIP), thereby inhibiting TGF-β1 degradation and sustaining pro-fibrotic signaling, which further broadens the mechanistic link between lactate accumulation and TGF-β pathway activation [83]. Moreover, in the context of diabetic nephropathy, H3K18la has been shown to increase the expression of IL-1β and NLRP3, triggering a local inflammatory cascade that further exacerbates EndoMT [84]. Taken together, although some of these mechanisms have not yet been directly confirmed in cardiovascular tissues, the available evidence suggests that lactylation may facilitate cardiovascular remodeling by promoting EndoMT and reinforcing TGF-β1-related pro-fibrotic signaling.

Beyond the cardiovascular system, accumulating studies in lung, liver, and kidney fibrosis further indicate that lactylation is broadly pro-fibrotic across organs, while the upstream triggers and cell-type targets appear to be context-dependent. In pulmonary fibrosis models, lactylation has been implicated in (a) direct activation of fibroblasts toward a myofibroblast-like state [85–87], (b) promotion of epithelial–mesenchymal transition (EMT)-linked programs [88], and (c) modulation of macrophage inflammatory signaling [89], which can secondarily enhance fibroblast activation and ECM deposition. In liver fibrosis, lactylation-associated programs have been linked to hepatic stellate cell activation, a central driver of collagen production and scar formation [90–92]. In kidney fibrosis, lactylation has been associated with EMT-related pathways and with macrophage-to-myofibroblast transition, both contributing to interstitial matrix accumulation [93,94]. Together, these multi-organ observations underscore the substantial contribution of lactylation to the progression of fibrosis.

### Lactylation in energy metabolism

CVDs are inherently energy-dependent, and lactate is one of the most sensitive metabolic indicators under hypoxic conditions [95,96]. Lactylation, as both a product of metabolic reprogramming and a mechanism that drives it, can reshape both glycolysis and mitochondrial metabolism, creating a metabolic feedback loop (Fig. 3B).

Lactylation has been shown to regulate OXPHOS by modulating key mitochondrial enzymes. As previously discussed, lactylation of MDH2 at Lys^241^ in myocardial I/R injury and of ATP5F1A at Lys^531^ in aortic dissection both impair enzyme activity, disrupting OXPHOS, and ultimately reducing ATP production [64,65]. In aortic aneurysm and dissection (AAD), elevated H4K16la promotes the transcription of pyruvate dehydrogenase kinase 1 (PDK1), thereby inhibiting pyruvate dehydrogenase (PDH) and shifting glucose-derived pyruvate away from mitochondrial oxidation toward lactate production in VSMCs [97]. Additionally, under hypoxic stress, cells, particularly muscle cells, show increased expression of AARS2, which catalyzes lactylation at Lys^457/458^ of CPT2 and at Lys^336^ of PDHA1 [98]. The resultant lactylation inactivates these 2 enzymes, thereby restricting acetyl-CoA production from pyruvate and fatty acid oxidation, ultimately suppressing OXPHOS.

In contrast to its impact on mitochondrial metabolism, lactylation also influences glycolytic pathways. Several studies have shown that histone lactylation (e.g., H3K9la and H4K12la) up-regulates glycolysis-associated genes, including HIF-1α, PKM2, and LDHA, which drive the shift toward a glycolytic phenotype [99–101]. However, lactylation of key glycolytic enzymes can have a suppressive effect. For example, lactylation of ALDOA, PKM2, and PFKP has been shown to inhibit glycolysis, suggesting that lactylation can both promote and inhibit glycolytic flux depending on the context and the specific target [102–105]. These findings highlight lactylation as a pivotal regulator of energy metabolism, balancing the demands of glycolysis and mitochondrial function in response to metabolic stress.

### Lactylation in immune and inflammatory responses

The immune and inflammatory responses are fundamental biological processes that maintain tissue homeostasis and protect the host from pathogens. In CVDs, these responses are not merely bystanders but active drivers of lesion evolution and tissue repair, requiring immune cells to rapidly adapt to changing microenvironmental cues [106]. Among these immune populations, macrophages are especially prominent orchestrators of cardiovascular inflammation and post-injury remodeling, given their abundance within lesions and their capacity for dynamic functional switching [107]. The phenotypic switch in macrophages, particularly between M1 (inflammatory) and M2 (reparative) states, is central to determining the fate of cardiovascular tissues in response to injury. Emerging evidence further indicates that metabolic reprogramming of macrophages critically shapes their activation states and effector functions in CVDs [108].

In line with this immunometabolic remodeling, lactate accumulation driven by enhanced glycolysis gives rise to protein lactylation, which has recently been recognized as a key regulator of macrophage phenotypic reprogramming in response to polarizing stimuli (Fig. 3C). For example, Irizarry-Caro et al. [109] demonstrated that the Toll-like receptor (TLR) signaling adapter BCAP promotes the inflammatory-to-reparative macrophage transition by enhancing histone lactylation. Mechanistically, elevated H3K18 lactylation up-regulates reparative genes (e.g., ARG1, KLF4, VEGFA, and IL-10), thereby restoring cardiac immune homeostasis and function [52,110]. Furthermore, increased H3K18 lactylation can induce expression of the m6A demethylase ALKBH5, which subsequently reduces methylation of NLRP3 mRNA and suppresses inflammasome activation [111]. Beyond histone modifications, lactylation of nonhistone proteins in macrophages, such as PKM2 and Mecp2, also contributes to M2 polarization [105,112].

Conversely, lactylation has been implicated in shifting macrophage polarization toward the pro-inflammatory M1 phenotype. For instance, in the type 2 diabetic myocardium, elevated H4K12 lactylation activates HIF-1α, thereby promoting pro-inflammatory polarization [113]. Similarly, in abdominal aortic aneurysms, H3K18 lactylation mediates GLI3 transcription, driving macrophage M1 polarization and amplifying the inflammatory response [114]. Moreover, lactate also promotes the secretion of damage-associated molecular patterns from macrophages, further enhancing inflammation [114]. In polymicrobial sepsis, intracellular lactate facilitates HMGB1 lactylation and acetylation. These modifications collectively drive exosomal HMGB1 secretion and increase endothelial permeability—key pathological features that exacerbate sepsis [73]. Taken together, lactylation serves as a fundamental and context-dependent mechanism that governs macrophage functional plasticity, influencing their polarization state and secretory profile across diverse cardiovascular and inflammatory diseases.

### Lactylation in angiogenesis

Angiogenesis is the process by which new blood vessels sprout from preexisting vasculature to meet increased demands for tissue perfusion and oxygen delivery [115]. In CVD, angiogenesis can be a double-edged sword: Neovascularization in ischemic myocardium may improve perfusion and support tissue repair, whereas intraplaque neovessels in atherosclerotic lesions can promote plaque vulnerability and increase the risk of hemorrhage [116,117]. Accumulating evidence indicates that lactylation constitutes an important regulatory layer of angiogenic signaling and thereby modulates disease progression (Fig. 3D). As the central pro-angiogenic cue, VEGF induces H3K9 lactylation in endothelial cells. This histone modification enriches the promoters of key angiogenic genes, such as TGFBR2, PRCP, and EGFR, thereby driving their transcriptional activation and promoting angiogenesis [118]. In the context of diabetic MI, the combined high-glucose and hypoxic microenvironment induce lactylation of IDH2 at Lys^272^ in endothelial cells, which enhances its binding to caveolin-1 (Cav1). This, in turn, relieves the constitutive inhibition of endothelial nitric oxide synthase (eNOS) by Cav1, leading to increased eNOS activity and nitric oxide (NO) production, ultimately facilitating cardiac microvascular endothelial proliferation, migration, and angiogenesis [38].

Ocular neovascularization disorders, a leading cause of blindness, are also regulated by lactylation. In endothelial cells, HIF-1α lactylation has been reported to enhance VEGFA up-regulation, stimulating angiogenesis [119]. Furthermore, H3K18 lactylation up-regulates the RNA methyltransferase NSUN2, which enhances m^5^C methylation on AKAP2 mRNA, increasing its stability. This cascade ultimately activates the protein kinase A (PKA)–VEGFR2 signaling pathway in endothelial cells, thereby promoting the development of choroidal neovascularization (CNV) [120]. Within the pathological milieu of diabetic retinopathy (DR), H3K18la modulates the expression of the fat mass and obesity-associated protein (FTO); FTO, in turn, promotes angiogenesis by accelerating endothelial cell cycle progression and enhancing tip cell formation [121]. Additionally, Wang et al. [122] reported that hypoxia-induced lactylation of the transcription factor YY1 in microglia transcriptionally activates FGF2 expression, contributing to pathological retinal neovascularization. Adequate blood supply is crucial for tumor growth, invasion, and metastasis. Research indicates that lactylation also contributes to cancer progression by supporting angiogenesis and promoting vasculogenic mimicry (VM) [123,124]. Taken together, these findings underscore the multifaceted regulatory role of lactylation in angiogenesis—targeting key molecules such as histones (H3K9 and H3K18), HIF-1α, YY1, and IDH2—and its contribution to pathological processes including diabetic MI, ocular neovascularization disorders, and cancer progression.

### Expanded biological functions of protein lactylation

In addition to the functions described above, lactylation participates in several other biological processes. For instance, Xing and colleagues [125] reported that the transcription factor Glis1 up-regulates glycolysis, thereby promoting H3K27 acetylation and H3K18 lactylation; these epigenetic modifications activate the pluripotency-associated genes OCT4 and SALL4 and facilitate somatic cell reprogramming. In a separate study, Dai et al. [126] demonstrated that lactate enhances myogenesis through p300-dependent H3K9la, a modification that stimulates NEU2 transcription to promote myoblast differentiation and muscle regeneration. In addition, Chen et al. [127] identified lactylation as a critical regulator of homologous recombination (HR) repair: Following DNA damage, the CBP acetyltransferase catalyzes K673 lactylation of MRE11, a core subunit of the MRN complex. This modification enhances the DNA-binding affinity of MRE11 and accelerates DNA end resection, thereby promoting HR. Collectively, these findings broaden the functional landscape of lactylation, positioning it as a versatile regulator of cell fate decisions, tissue regeneration, and genome maintenance.

## Mechanism and Role of Lactylation in CVDs

**Fig. 4.**
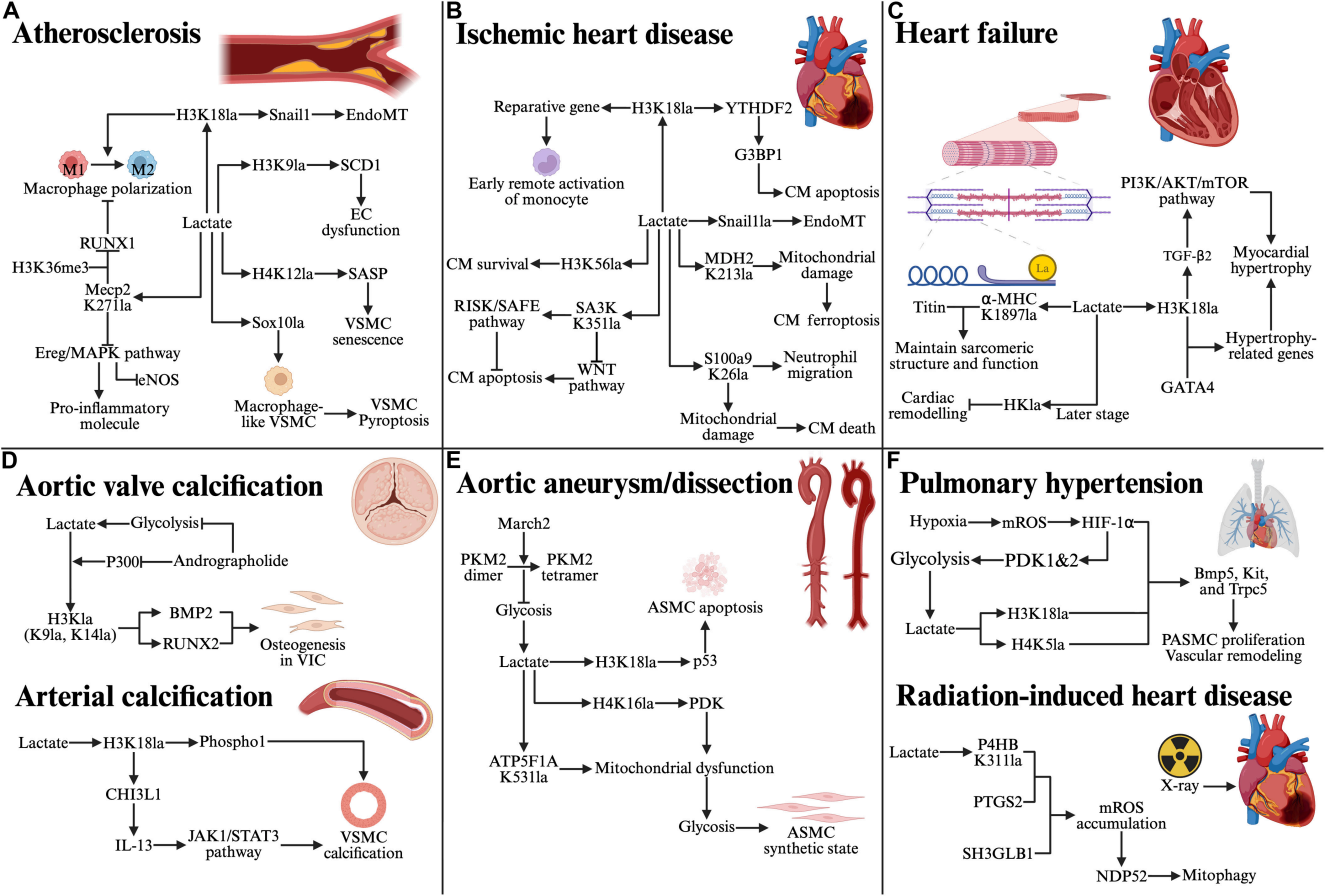
Mechanisms of lactylation in CVDs. Lactate-driven lactylation modulates histone/nonhistone targets to shape cardiovascular pathology. In (A) to (C), the left subpanels depict protective (“brake”) lactylation that suppresses disease progression, whereas the right subpanels depict deleterious (“accelerator”) lactylation that promotes disease progression. In contrast, (D) to (F) summarize pro-disease lactylation mechanisms. (A) Atherosclerosis. Protective lactylation in macrophage favors M2 polarization and plaque stabilization; deleterious lactylation in endothelial cell (EC)/VSMC drives EndoMT, endothelial dysfunction, VSMC senescence, and VSMC pyroptosis. (B) Ischemic heart disease. Protective lactylation supports reparative gene programs and pro-survival signaling; deleterious lactylation promotes EndoMT, cardiomyocyte apoptosis/ferroptosis, and neutrophil recruitment-linked injury. (C) Heart failure. Protective lactylation preserves sarcomeric integrity and can be beneficial at later stages; deleterious H3K18la activates pro-hypertrophic signaling (e.g., TGF-β/PI3K/AKT/mTOR and GATA4-dependent transcription). (D) Aortic valve calcification: Lactate-driven histone lactylation promotes VIC osteogenic differentiation and valve calcification; andrographolide alleviates calcification by suppressing this lactate–lactylation axis. Arterial calcification: Histone lactylation induces pro-calcific programs to accelerate VSMC calcification. (E) Aortic aneurysm/dissection: Enhanced glycolysis/lactate and lactylation promotes VSMC apoptosis, mitochondrial dysfunction, and phenotypic switching toward a synthetic state, thereby driving aneurysm/dissection progression. (F) Pulmonary hypertension: Hypoxia-induced lactate accumulation and histone lactylation promote PASMC proliferation and vascular remodeling. Radiation-induced heart disease: P4HB lactylation promotes mROS accumulation and NDP52-dependent mitophagy after irradiation. Created in BioRender. 1, 1. (2026) https://BioRender.com/uu23pus.

Within the pathophysiology of cardiovascular disorders, lactylation has been identified as an important epigenetic regulatory mechanism. Accumulating studies have identified a panel of CVD-associated lactylation events on both histone and nonhistone proteins, providing an evidence base for disease relevance (Table 1). The mechanistic pathways through which these modifications operate, together with their functional consequences across distinct cardiovascular conditions, are delineated in the following subsections (Fig. 4).

**Table 1.** CVD-associated lactylated proteins

Lactylated protein	Site	Writer/eraser	CVD context	Cell type	Effect on CVD	Reference
Histone H3	K9, K18	p300/NA	Atherosclerosis	Endothelial cell	Detrimental	[57,62]
Histone H4	K12	NA/HDAC3	Atherosclerosis	Vascular smooth muscle cell	Detrimental	[63]
Sox10	NA	NA/NA	Atherosclerosis	Vascular smooth muscle cell	Detrimental	[80]
Histone H3	K18	p300/NA	Atherosclerosis	Macrophage	Protective	[128]
Mecp2	K271	p300/HDAC3	Atherosclerosis	Macrophage	Protective	[112]
Mecp2	K271	p300/NA	Atherosclerosis	Endothelial cell	Protective	[129]
Snail	NA	p300/NA	Myocardial infarction	Endothelial cell	Detrimental	[78]
Histone H3	K18	p300/NA	Myocardial ischemia/reperfusion injury	Cardiomyocyte	Detrimental	[58]
MDH2	K241	NA/NA	Myocardial ischemia/reperfusion injury	Cardiomyocyte	Detrimental	[64]
S100a9	K26	DLAT/NA	Myocardial ischemia/reperfusion injury	Neutrophil	Detrimental	[37]
Histone H3	K56	p300/NA	Myocardial ischemia/reperfusion injury	Cardiomyocyte	Protective	[132]
Histone H3	K18	p300, GCN5/NA	Myocardial ischemia/reperfusion injury	Macrophage	Protective	[52]
Serpina3k	K351	NA/NA	Myocardial ischemia/reperfusion injury	Fibroblast	Protective	[70]
Histone H3	K18	P300, GCN5/NA	Heart failure	Cardiomyocyte	Detrimental	[59]
α-MHC	K1897	p300/SIRT1	Heart failure	Cardiomyocyte	Protective	[66]
Histone H3	K9, K14	p300/NA	Calcific aortic valve disease	Valve interstitial cell	Detrimental	[143,144]
Histone H3	K18	p300/NA	Arterial calcification	Vascular smooth muscle cell	Detrimental	[60,145]
Histone H3	K18	NA/NA	Aortic aneurysm and dissection	Macrophage	Detrimental	[114]
Histone H3	K18	NA/NA	Aortic aneurysm and dissection	Vascular smooth muscle cell	Detrimental	[61]
Histone H4	K16	HBO1/NA	Aortic aneurysm and dissection	Vascular smooth muscle cell	Detrimental	[97]
ATP5F1A	K531	NA/SIRT3	Aortic aneurysm and dissection	Vascular smooth muscle cell	Detrimental	[65]
Histone H3	K18	NA/NA	Pulmonary hypertension	Vascular smooth muscle cell	Detrimental	[147]
P4HB	K31	NA/NA	Radiation-induced cardiac injury	Vascular smooth muscle cell	Detrimental	[67]

NA, not available

### Lactylation in atherosclerosis

Atherosclerosis is a major risk factor underlying the development and progression of CVDs, characterized fundamentally as a lipid metabolism-driven chronic inflammatory disorder. Endothelial cells, VSMCs, and macrophages are widely recognized as key cellular players driving disease initiation, progression, and plaque stability. Recent studies have revealed that lactylation serves as a crucial epigenetic regulator in atherosclerosis, modulating these cell populations through distinct pathways (Fig. 4A).

In endothelial cells, current evidence indicates that p300-mediated histone lactylation at 2 specific sites plays an essential role in promoting atherosclerosis. H3K18 lactylation promotes EndoMT through transcriptional activation of Snail1, thereby exacerbating endothelial dysfunction and ECM remodeling [57]. Meanwhile, H3K9 lactylation transcriptionally activates stearoyl-CoA desaturase 1 (SCD1), promoting a pro-inflammatory endothelial dysfunction characterized by impaired endothelium-dependent vasodilation, up-regulation of adhesion molecules, and increased monocyte adhesion [62]. In VSMCs, 2 distinct lactylation-dependent pathways contribute to atherosclerosis. First, Sox10 lactylation promotes macrophage-like transdifferentiation and pyroptosis, thereby intensifying vascular inflammation and neointimal hyperplasia [80]. Second, TRAP1-mediated metabolic reprogramming induces a glycolytic shift, increasing lactate production, which in turn induces H4K12la through HDAC3 down-regulation [63]. Binding of H4K12la to promoters of senescence-associated secretory phenotype (SASP) genes accelerates cellular senescence and plaque formation.

By contrast, in macrophages, lactylation can be linked to repair-oriented programs and plaque stabilization. Inhibition of MCT4 enhances p300-mediated H3K18la, which up-regulates reparative and TCA cycle-related genes, thereby promoting M2 polarization and supporting plaque stabilization [128]. Furthermore, Chen et al. [112] demonstrated that exercise serves as a key trigger for lactylation-mediated regulation in atherosclerosis, conferring cardioprotective effects. Mechanistically, exercise-induced Mecp2 lactylation at Lys^271^ (Mecp2 K271la) in macrophages interacts with H3K36me3 to repress RUNX1 transcription, thereby driving M2 polarization and contributing to plaque stabilization. In addition, exercise promotes Mecp2 K271la in endothelial cells, which attenuates atherogenesis by inhibiting the Ereg/mitogen-activated protein kinase (MAPK) signaling axis. Consequently, pro-inflammatory molecules (IL-1β, IL-6, and VCAM-1) are down-regulated, whereas eNOS is up-regulated [129].

Overall, lactylation exerts dual effects in atherosclerosis, influencing disease progression through diverse mechanisms in these key cellular players. Within this “accelerator–brake” framework, lactylation in endothelial cells and VSMCs predominantly accelerates pro-sclerotic, pro-inflammatory, and EndoMT/VSMC-transdifferentiation programs, whereas MeCP2- and H3K18-centered lactylation in macrophages can function as a brake by promoting reparative M2 polarization and plaque stabilization. These cell type-specific and directionally opposing effects underscore the therapeutic potential of modulating lactylation to restore vascular homeostasis in atherosclerosis.

### Lactylation in MI and reperfusion

MI, a leading cause of mortality worldwide, results from the accumulation of coronary plaques that severely restrict coronary blood flow and induce profound hypoxia. Timely coronary reperfusion remains the standard of care for MI; however, it can paradoxically precipitate myocardial ischemia/reperfusion injury (MIRI), thereby amplifying cardiac damage [130]. Cardiac ischemia induces profound metabolic remodeling: Hypoxia impairs mitochondrial OXPHOS, resulting in ATP depletion that fails to meet myocardial energy demands. Consequently, metabolic reprogramming increases glycolytic flux as the principal ATP-generating pathway, causing marked lactate accumulation in ischemic myocardial tissue [131]. High lactate concentrations are strongly associated with worse clinical outcomes and increased mortality in patients with MI. At the molecular level, lactylation functions as a key regulatory link between ischemia-driven lactate accumulation and downstream cardiac injury responses (Fig. 4B).

In maladaptive remodeling, the post-MI surge in lactate induces Snail1 lactylation, thereby promoting EndoMT, aggravating cardiac fibrosis, and impairing cardiac function [78]. In cardiomyocytes, I/R-induced histone H3K18 lactylation up-regulates YTHDF2, which in turn promotes apoptosis via Ras guanosine triphosphatase (GTPase)-activating protein-binding protein 1 (G3BP1), thereby exacerbating post-ischemic remodeling [58]. Simultaneously, MDH2 lactylation impairs mitochondrial function and drives iron-dependent lipid peroxidation, thereby depleting glutathione (GSH), inactivating glutathione peroxidase 4 (GPX4), and ultimately inducing ferroptosis in cardiomyocytes [64]. Importantly, S100a9 lactylation at Lys^26^ (S100a9 K26la) in neutrophils can further amplify injury. Mechanistically, S100a9K26la translocates to the nucleus and acts as a co-activator at promoters of migration-related genes, promoting neutrophil trafficking and recruitment to ischemic myocardium; moreover, during neutrophil extracellular trap (NET) formation, lactylated S100a9 can be released locally and induce cardiomyocyte death through mitochondrial dysfunction [37].

Recent studies exploring the cardioprotective effects of protein lactylation have highlighted its context-dependent roles in the pathophysiology of MI and MIRI. In the early phase of MI, endogenous glycolytic reprogramming coupled with lactate import via MCT1 enhances histone lactylation within bone marrow and circulating monocytes [52]. Specifically, H3K18la facilitates transcription of reparative genes such as LRG1, VEGFA, and IL-10, thereby establishing a pro-reparative microenvironment that improves cardiac function following MI/MIRI [52]. In cardiomyocytes, Yu et al. [132] reported that HSPA12A is crucial for improving cardiomyocyte survival during I/R by maintaining aerobic glycolytic homeostasis and sustaining H3K56 lactylation. Additionally, fibroblasts stimulated by I/R secrete lactylated Serpina3k (SA3K), which limits cardiomyocyte apoptosis through paracrine mechanisms. SA3K lactylation at Lys^351^ (SA3K K351la) inhibits Wnt signaling while activating 2 principal pro-survival cascades: (a) the RISK (reperfusion injury salvage kinase) pathway through phosphorylation of AKT and extracellular signal-regulated kinase (ERK1/2), and (b) the SAFE (survivor activating factor enhancement) pathway via Janus kinase 2 (JAK2)–signal transducer and activator of transcription 3 (STAT3) signaling [70].

Collectively, these findings demonstrate that lactylation exerts spatiotemporally distinct and often opposing roles in MI and MIRI. On the one hand, it can act as an accelerator of injury by driving maladaptive processes such as EndoMT, cardiomyocyte apoptosis, and ferroptosis, thereby exacerbating tissue damage and adverse remodeling. On the other hand, lactylation in monocytes, macrophages, and fibroblasts forms a compensatory brake that supports angiogenesis, resolution of inflammation, and tissue repair. This dual regulatory network underscores the context-dependent nature of lactylation in cardiac pathophysiology. Therapeutic modulation of protein lactylation may therefore represent a promising strategy for alleviating ischemic myocardial injury and promoting cardiac repair.

### Lactylation in heart failure

As a complex clinical syndrome, heart failure arises from structural and/or functional abnormalities of the myocardium that impair ventricular performance, resulting in insufficient cardiac output and/or elevated intracardiac pressures [133]. These pathophysiological changes lead to systemic or pulmonary congestion and end-organ hypoperfusion. Analogous to MI, the failing heart undergoes metabolic reprogramming, characterized by increased glycolysis and reduced fatty acid oxidation [95]. Elevated blood lactate concentrations serve as a robust prognostic marker, predicting worse clinical outcomes and a higher incidence of adverse cardiovascular events [134]. In cardiomyocytes, lactate contributes to pathological cardiac hypertrophy through histone lactylation, particularly H3K18la [135] (Fig. 4C). H3K18la up-regulates TGF-β2 expression and activates the phosphatidylinositol 3-kinase ((PI3K)/AKT/mammalian target of rapamycin (mTOR) signaling pathway, thereby promoting pressure overload-induced pathological myocardial hypertrophy [59]. Moreover, H3K18la interacts with the transcription factor GATA4 to enhance its transcriptional activity, which in turn activates the expression of hypertrophy-related genes (such as ANP, BNP, and β-MHC) and ultimately accelerates heart failure progression [136]. However, in advanced heart failure, up-regulation of MCT4 and enhanced lactate efflux are prominent [137]. Under this condition, maintaining histone lactylation has been shown to attenuate disease progression and improve cardiac function [138]. Additionally, Zhang et al. [66] demonstrated that loss of lactylation at the K1897 site of α-MHC impairs its binding to titin, leading to deterioration of cardiac structure and function.

Collectively, these findings suggest a context-dependent duality of lactylation in heart failure. While early-stage histone lactylation appears to drive pathological hypertrophy and disease progression, the preservation of lactylation at later stages, including on α-MHC, may exert protective effects on sarcomeric integrity and cardiac function. This temporal shift implies that lactylation functions not as a simple “accelerator” or “brake”, but as a dynamic modifier whose role depends on the cellular metabolic milieu and disease phase. Importantly, the regulatory capacity of lactylation highlights its potential as a therapeutic target; modulating specific lactylation sites or levels could offer novel strategies to decelerate maladaptive remodeling and improve cardiac outcomes in heart failure.

### Lactylation in valve and arterial calcifications

Cardiovascular calcification is a common pathological feature affecting both heart valves and arteries, typically initiating insidiously and progressing over time, particularly in aging populations and chronic disease states. Calcific aortic valve disease (CAVD), a prototypical form of valvular calcification, is characterized by progressive valve tissue thickening, sclerosis, and the formation of calcified nodules, which collectively culminate in valvular stenosis and a spectrum of clinical sequelae, including coronary insufficiency, dyspnea, and angina [139,140]. By contrast, arterial calcification involves abnormal calcium deposition within the arterial wall, a process that impairs vascular compliance and elasticity and shows strong associations with multiple cardiovascular disorders [141,142]. Recent studies have begun to elucidate the role of lactylation in regulating these calcification processes, as summarized below (Fig. 4D). In the context of aortic valve calcification, lumican—an ECM component—has been identified as a key contributor to CAVD pathogenesis by promoting histone H3 lactylation [143]. Mechanistically, lumican activates inflammatory responses and enhances glycolysis in valve interstitial cells (VICs), leading to substantial lactate accumulation. The resulting lactate subsequently induces site-specific H3Kla, particularly at the H3K14 and H3K9 residues; this epigenetic modification up-regulates the transcription of calcification-related genes, including RUNX2 and BMP2, thereby accelerating valvular calcification. Moreover, andrographolide (AGP) has been shown to attenuate CAVD progression by simultaneously modulating glycolytic pathways and inhibiting p300-mediated H3Kla, highlighting a potential therapeutic strategy for mitigating valvular calcification [144]. With respect to arterial calcification, Ma et al. [60] were the first to report that the orphan nuclear receptor NR4A3 is up-regulated in calcified arterial tissues. Subsequent investigations further demonstrated that NR4A3 directly binds to the promoters of glycolytic genes, thereby augmenting lactate production in VSMCs. The increased lactate levels in turn drive histone lactylation—most notably H3K18la—which promotes arterial medial calcification by activating Phospho1 expression [60]. Furthermore, microenvironmental lactate accumulation exacerbates VSMC calcification through H3K18la-mediated up-regulation of CHI3L1*,* which activates the IL-13/IL-13Rα2/JAK1/STAT3 signaling cascade and thereby aggravates diabetes-related arterial calcification [145]. Collectively, these findings highlight lactylation—positioned at the interface of glycolytic metabolism and histone modification—as a central regulator of both valvular and arterial calcification and point to lactylation-related pathways as promising therapeutic targets for prevalent CVDs.

### Lactylation in AAD

AAD are highly lethal cardiovascular emergencies with limited pharmacologic options beyond surgical/interventional repair, and disease progression is tightly linked to VSMC phenotypic switching from a contractile to a synthetic state [146]. Growing evidence indicates that this switch is accompanied by metabolic reprogramming resulting in lactate accumulation and mitochondrial dysfunction in the aortic wall. Accumulating evidence further underscores the regulatory role of protein lactylation in AAD pathogenesis (Fig. 4E). In AAD, studies have reported down-regulated expression of the E3 ubiquitin ligase March2 in VSMCs. Further investigation reveals that March2 deficiency impairs PKM2 polymerization, thereby promoting glycolysis and enhancing H3K18 lactylation, which drives p53-dependent apoptosis of VSMCs [61]. In parallel, lactylation marks have been implicated in reinforcing the phenotypic switch: Elevated H4K16 lactylation has been linked to increased transcription of PDK1, which further biases cellular metabolism toward glycolysis, forming a positive feedback loop that sustains metabolic reprogramming [97]. Moreover, ATP5F1A lactylation in VSMCs further exacerbates mitochondrial dysfunction [65]. These metabolic alterations drive the phenotypic switching of VSMCs toward a matrix-degrading synthetic state, thereby promoting pathological vascular remodeling and AAD progression [65].

### Lactylation in other cardiovascular-related diseases

Alongside the diseases discussed earlier, lactylation has also been linked to hypoxic pulmonary hypertension and radiation-induced cardiac injury (Fig. 4F). In hypoxic pulmonary hypertension, the mitochondrial reactive oxygen species (mROS)/HIF-1α/PDK1/2 axis induces glycolytic reprogramming, promoting lactate accumulation and histone lactylation at proliferation-related genes and leading to hyperplasia of pulmonary artery smooth muscle cells (PASMCs) [147]. Radiation-induced cardiac injury is associated with Lys^311^ lactylation of protein disulfide-isomerase (P4HB). This modification promotes the interaction of P4HB with PTGS2, thereby enhancing SH3GLB1-mediated mitochondrial ROS (mitoROS)-driven mitophagy, which can be inhibited by aloe emodin through site-specific blockade of P4HB K311 lactylation [67].

## Potential Lactylation-Targeted Therapies for CVDs

Therapeutic targeting of lactylation in CVDs can be conceptualized within a 3-ring metabolic–epigenetic framework along the lactate–lactylation axis. In this framework, the outer ring focuses on modulating lactate transport, primarily through monocarboxylate transporters such as MCT1 and MCT4; the middle ring targets lactate production by glycolytic enzymes, including LDHA and PKM2; and the inner ring directly modulates lactylation-modifying enzymes, including writers and erasers such as p300/CBP, other HATs, HDACs, and sirtuins. These concentric layers represent progressively more proximal interventions on the lactylation machinery and collectively define a translational roadmap for small-molecule and combination therapies in CVDs. Accumulating evidence indicates that a variety of small molecules and approved pharmaceuticals can effectively regulate intracellular lactate and lactylation levels (Table 2), with their mechanisms largely corresponding to these 3 rings.

**Table 2.** Molecules and drugs targeting lactate metabolism or lactylation

Mechanism	Molecule/drug	Target	Research status	Disease	Reference
Lactate transporters	AZD3965	Inhibits MCT1	Clinical trial	Acute pancreatitis	[156]
	CHC	Inhibits MCT1	Preclinical	Sepsis-induced acute lung injury	[157]
	AR-C122982	Inhibits MCT1	Preclinical	Cervical cancer	[158]
	AR-C155858	Inhibits MCT1/2	Preclinical	NA	[159]
	Syrosingopine	Inhibits MCT1/4	Preclinical	NA	[159]
	VB124	Inhibits MCT4	Preclinical	Atherosclerosis	[128]
Lactate production	Galloflavin	Inhibits LDH	Preclinical	Bladder cancer	[160]
	FX-11	Inhibits LDHA	Preclinical	Ovarian cancer	[161]
	GSK2837808A	Inhibits LDHA	Preclinical	Nonalcoholic fatty Liver disease	[162]
	Oxamate	Inhibits LDHA	Preclinical	Glioblastoma	[163]
	GNE-140	Inhibits LDHA	Preclinical	NA	[164]]
HATs	C646	Inhibits p300	Preclinical	Pancreatic ductal Adenocarcinoma	[165]
	A485	Inhibits p300/CBP	Preclinical	Retinopathy of prematurity	[122]
	MG149	Inhibits TIP60	Preclinical	Colorectal cancer	[166]
	WM3835	Inhibits HBO1	Preclinical	Aortic aneurysm and dissection	[97]
HDACs	ITSA-1	Activates HDAC	Preclinical	Atherosclerosis	[63]
	TSA	Inhibits HDAC class I/II	Clinical trial	Gastric cancer	[167]
	MS-275	Inhibits HDAC class I	Clinical trial	Head and neck squamous cell carcinoma	[168]
	Tucidinostat	Inhibits HDAC class I	Approved drug	Triple-negative breast cancer	[169]
	SRT1720	Activates SIRT1	Preclinical	Heart failure	[66]
	EX527	Inhibits SIRT1	Preclinical	Heart failure	[66]
	AGK2	Inhibits SIRT2	Preclinical	Gastric cancer	[45]
	Honokiol	Activates SIRT3	Clinical trial	Hepatocellular carcinoma	[170]]
	3-TYP	Inhibits SIRT3	Preclinical	Sepsis-induced acute kidney injury	[171]

NA, not available

### Outer ring: Targeting lactate transporters

Interventions targeting MCT1 and MCT4 constitute the outer ring of this framework. As key mediators of lactate transport in vivo, MCTs are critical for maintaining intracellular and extracellular lactate equilibrium. In angiotensin II (Ang II)-induced models of hypertensive cardiac remodeling, a distinct intercellular lactate exchange mechanism operates: Cardiac fibroblasts predominantly export lactate through MCT4, whereas cardiomyocytes take it up via MCT1 [148]. This lactate shuttle markedly up-regulates the expression of genes associated with cardiomyocyte hypertrophy (e.g., ANP, BNP, and MYH7) and accelerates the progression of pathological cardiac remodeling. Accordingly, cardiomyocyte-specific deletion of MCT1 attenuates Ang II-induced cardiac hypertrophy and fibrosis by impairing lactate uptake [148]. Moreover, the selective MCT4 inhibitor VB124 mitigates cardiomyocyte hypertrophy and improves cardiac remodeling by inhibiting lactate export and rebalancing the pyruvate–lactate axis toward mitochondrial pyruvate oxidation [149]. Consistently, inhibiting lactate efflux in cardiomyocytes elevates intracellular lactate and restores α-MHC K1897 lactylation, thereby preserving sarcomeric integrity and alleviating heart failure progression [66]. Collectively, these findings underscore the therapeutic potential of MCT inhibitors as promising targets for CVDs.

### Middle ring: Targeting lactate production

As the middle ring of the framework, this strategy focuses on limiting the generation of the lactate pool that fuels protein lactylation. In practice, this strategy is primarily achieved by inhibiting key glycolytic enzymes, particularly LDHA. Evidence for this relationship is derived from cerebral I/R injury models [150]. In N2a cells undergoing oxygen–glucose deprivation/reoxygenation, silencing LDHA with small interfering RNA (siRNA) reduces lactate production, leading to decreased H3K18la levels and attenuated HMGB1-mediated pyroptosis. Conversely, exogenous lactate supplementation restores histone lactylation in these cells, thereby confirming the causal link between LDHA-derived lactate and this epigenetic modification [150]. Similarly, in cardiac models, hypoxia/reoxygenation up-regulates LDHA in H9c2 cells, increasing lactate and global lactylation [151]. In this context, LDHA silencing reduces lactate, diminishes NLRP3 K245 lactylation, inhibits pyroptosis, and mitigates myocardial injury. In addition, pharmacological inhibition of LDH with oxamate or the specific LDHA inhibitor GNE-140 also reduces intracellular lactate, suppresses H3K18 lactylation, and alleviates cardiomyocyte hypertrophy [135]. Together, LDHA acts as a central mediator of lactate-driven metabolic reprogramming, thereby presenting a novel therapeutic avenue for the treatment of CVDs.

### Inner ring: Targeting enzymes of lactylation

The inner ring provides the most proximal control by directly modulating lactylation-modifying enzymes, offering a powerful lever to rewire lactate-responsive gene programs. As previously established, lactylation is dynamically regulated by lactyltransferase “writers” and deacetylase “erasers”. The p300/CBP family are key lactyltransferases; their silencing or pharmacological inhibition (e.g., with C646) markedly diminishes lactylation in macrophages [73,89]. In vitro, AGP suppresses aortic valve calcification by disrupting p300-mediated H3K9 and H3K14 lactylation, thereby inhibiting RUNX2 expression and osteogenic differentiation in VICs [144]. HDAC1 to HDAC3 function as efficient erasers of lactylation. The HDAC agonist ITSA-1 potently inhibits Ras-induced H4K12 lactylation and senescence in VSMCs, producing effects comparable to those observed with HDAC3 overexpression [63]. Conversely, HDAC3 down-regulation enhances myocardial lactylation, alleviating cardiac remodeling and improving cardiac function [138]. Collectively, these findings identify p300/CBP and HDACs as tractable targets for cardiovascular therapy via modulation of lactylation, underscoring their substantial translational potential.

### Feasibility and translational considerations

The 3-ring metabolic–epigenetic framework provides a practical translational roadmap. However, the feasibility of lactylation-targeted therapies for CVDs depends on several critical factors that must be addressed to ensure successful clinical translation. First, the biological effects of lactylation are highly cell-type specific and stage dependent, meaning it can function as an “accelerator” or a “brake” depending on where and when it occurs. Even within the same disease, lactylation in endothelial cells, VSMCs, or immune cells (e.g., macrophages) may engage distinct transcriptional programs and thus either confer protection or exacerbate pathology; as the disease advances, its net effect may also switch direction. Accordingly, lactylation-targeted therapies should be tailored to both the cellular source and the disease stage, with consideration of temporally staged interventions that exploit appropriate therapeutic windows and dosing strategies. Second, targeted delivery to the heart and vasculature is paramount, as lactate transporters and lactylation-related enzymes are expressed in a variety of tissues, including muscle and brain [19]. Systemic inhibition may cause unwanted metabolic consequences, so novel drug delivery systems, such as nanoparticles or receptor-mediated targeting, will be essential to restrict the therapeutic effects to the heart and blood vessels [152]. Third, the development of pharmacodynamic biomarkers will be critical for monitoring the efficacy and safety of lactylation-targeted therapies. Site-specific biomarkers, such as lactylation on histones and nonhistone proteins, as well as transcriptional signatures, could provide real-time insights into target engagement and therapeutic responses [153]. Finally, safety considerations are paramount, as many cardiovascular patients are on long-term therapies for other conditions, such as hypertension or dyslipidemia. The potential for drug–drug interactions, especially with other epigenetic modulators or metabolic regulators, must be carefully assessed [154]. Translational success will depend on addressing these feasibility concerns, including optimizing patient selection, developing safe and effective delivery methods, and establishing reliable biomarkers to guide therapy.

## Perspectives

**Fig. 5.**
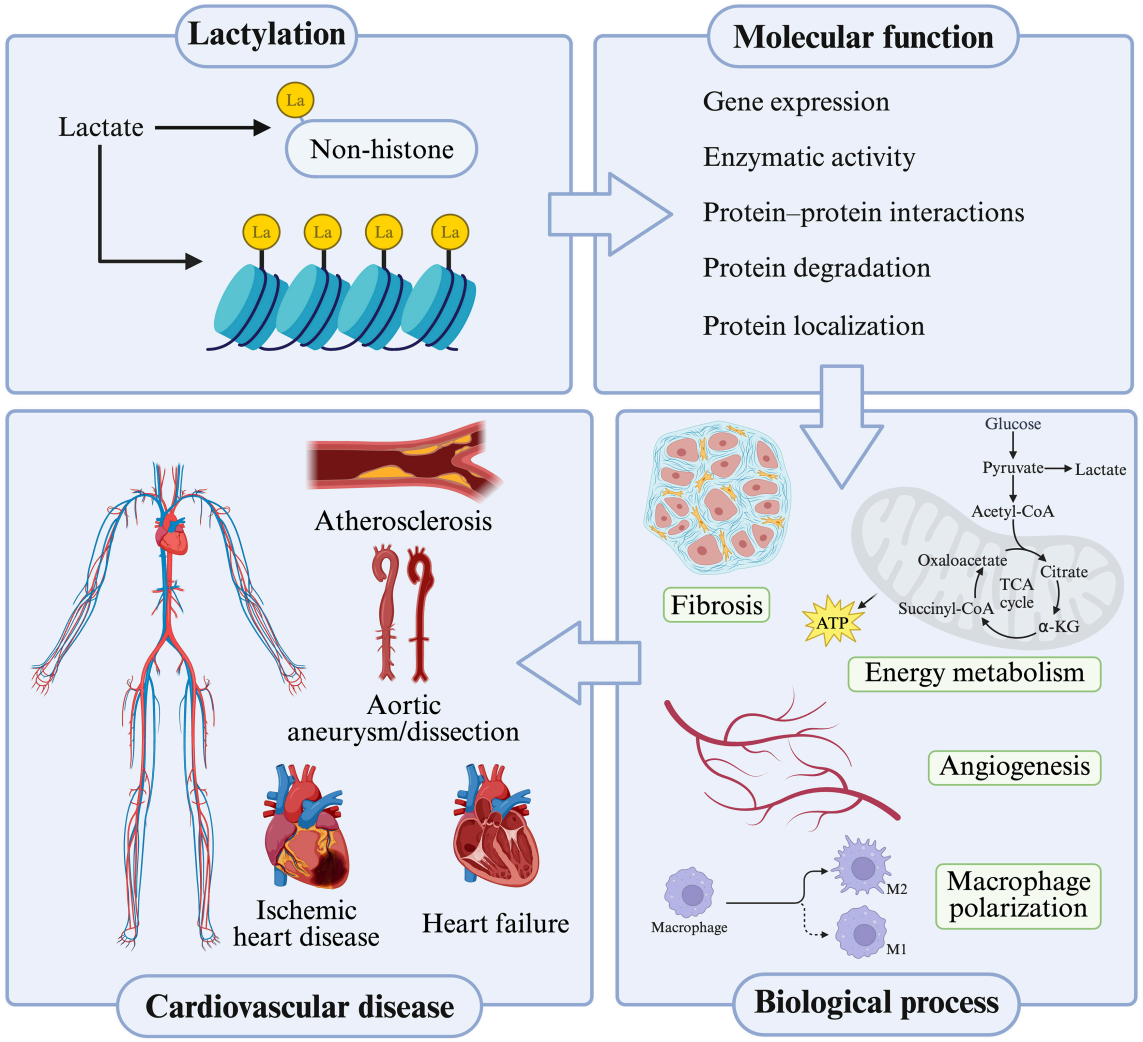
Overview of lactylation modification from basic biological processes to cardiovascular diseases. Lactate-derived lactylation occurs on both histone and non-histone proteins and regulates multiple molecular functions, including gene expression, enzymatic activity, protein–protein interactions, protein degradation, and protein localization. Through these molecular effects, lactylation modulates key biological processes relevant to cardiovascular homeostasis and pathology, such as fibrosis, energy metabolism, angiogenesis, and macrophage polarization. Collectively, these lactylation-associated mechanisms contribute to the development and progression of major cardiovascular diseases, including atherosclerosis, aortic aneurysm/dissection, ischemic heart disease, and heart failure. Created in BioRender. 1, 1. (2026) https://BioRender.com/0bmltfo.

As a recently identified PTM derived from lactate, lactylation has become a pivotal molecular link between metabolic reprogramming and epigenetic regulation. Accumulating evidence demonstrates that lactylation participates in a wide array of cellular processes, including inflammation, fibrosis, angiogenesis, and metabolism, many of which are central to the pathogenesis of CVDs [155]. In this regard, this review comprehensively and systematically summarizes recent advances in lactylation and highlights the multifaceted roles of protein lactylation in major CVDs, thereby supporting its promise as a disease biomarker and a potential therapeutic target (Fig. 5).

Despite these advances, several critical issues remain unresolved. First, although most current research focuses on l-lactylation, the specific contributions of d-lactylation to disease-related phenotypes remain incompletely understood. Second, at the chromatin level, crosstalk between lactylation and other acylations (acetylation, crotonylation, β-hydroxybutyrylation, succinylation), as well as with DNA/RNA methylation, remains largely correlative. Third, the mechanisms by which metabolic states and lactate concentrations dictate the functional consequences of lactylation within the same cell type remain unclear. Additionally, conceptualizing lactylation as a context-dependent “accelerator and brake” in cardiovascular pathology provides a useful framework for designing precision interventions. By integrating strategies that modulate lactate availability with agents that selectively target lactylation writers, readers, and erasers, it may be possible to develop a new generation of metabolism–epigenetics combination therapies for the prevention and treatment of CVDs. However, challenges related to tissue specificity, potential off-target effects, and the complexity of lactate signaling networks must be carefully addressed.

## Conclusion

In the cardiovascular field, lactylation has emerged as a novel yet pivotal focus of research at the interface of metabolism and gene regulation. Elucidating its molecular mechanisms and intermolecular interactions provides a fundamental basis for further investigation in this area. Such a foundation is indispensable for the development of future interventions that target the lactylation pathway. Furthermore, this foundation will facilitate the rigorous assessment of the efficacy and safety of newly developed interventions in preclinical and clinical settings. Therefore, advancing our understanding of lactylation is a crucial prerequisite for the rational design of precise and personalized treatments for CVDs.
